# *Stenotrophomonas maltophilia* in Salad

**DOI:** 10.3201/eid1107.040130

**Published:** 2005-07

**Authors:** Andleeb Qureshi, Louise Mooney, Miles Denton, Kevin G. Kerr

**Affiliations:** * University of Leeds, Leeds, United Kingdom;; † General Infirmary at Leeds, Leeds, United Kingdom;; ‡ Harrogate and District National Health Service Foundation Trust, Harrogate, United Kingdom;; § Hull York Medical School, York Campus, Heslington, United Kingdom

**Keywords:** food microbiology, Stenotrophomonas, immunocompromised patient

**To the Editor:** *Stenotrophomonas maltophilia* has emerged as an important nosocomial pathogen, especially in debilitated and immunocompromised persons (1). However, comparatively little is known of the epidemiology of this bacterium, and sources and routes of transmission of *S*. *maltophilia* are not well understood. The bacterium is widely distributed in the environment, including in plant rhizospheres (2). Although environmental sources such as ice-making machines have been implicated in outbreaks of nosocomial *S*. *maltophilia* sepsis, the source of infection in other outbreaks and in sporadic cases often remains unidentified. Food as a source of the bacterium has not been investigated; given the association of the bacterium with plants, we investigated the prevalence of the bacterium in salad vegetables.

Salads were purchased from reputable supermarkets and transported immediately to the laboratory. Ten grams of salad was homogenized in 90-mL sterile saline in a stomacher for 1 to 2 min. Aliquots (200 μL) of decimal dilutions of the homogenate were plated onto vancomycin-imipenem-amphotericin B (VIA) agar (3) and incubated for 24 to 48 h at 30°C. Occasional imipenem-resistant environmental bacteria, such as *Janthinobacterium lividum* or vancomycin-resistant *Enterococcus faecium*, may grow on VIA, but the medium contains a mannitol/bromothymol blue indicator system, allowing these bacteria, which produce acid from mannitol, to be distinguished from *S*. *maltophilia*. Putative *S*. *maltophilia* colonies were further identified by the API 20NE system (bioMérieux, Marcy l'Etoile, France).

Susceptibilities of 9 confirmed isolates to ceftazidime, chloramphenicol, colistin sulfate, gentamicin, minocycline, piperacillin/tazobactam, and trimethoprim-sulfamethoxazole were determined by using a disk diffusion method. *Pseudomonas aeruginosa* (NCTC10662) was used as a control. Because disk diffusion is not a reliable method for determining the susceptibility of *S*. *maltophilia* to quinolone antimicrobial agents (1), the Etest was used.

*S. maltophilia* was cultured from 14 (78%) of 18 salads. Numbers ranged from 1.50 × 10^2^ to 1.96 × 10^5^ CFU/g (mean 1.75 × 10^5^ CFU/g, median 7.05 × 10^3^ CFU/g). All isolates were susceptible to ciprofloxacin, colistin sulfate, minocycline, and trimethoprim-sulfamethoxazole. Eight (89%) were resistant to chloramphenicol; 7 (78%) to piperacillin/tazobactam; 5 (56%) to ceftazidime, and 2 (22%) to gentamicin.

All products examined were labeled "washed and ready to eat," and, thus, consumers would be unlikely to wash these products before consumption. The growth characteristics of *S*. *maltophilia* in products of this type, especially if subject to temperature abuse, are unknown, but in the domestic setting, numbers of the bacterium may increase before use.

All of the 9 isolates examined exhibited resistance to >2 of the 8 agents tested, with 2 isolates resistant to 4 compounds and thus had resistance phenotypes similar to those of strains associated with human infection. These findings are in agreement with those of Berg (2), who reported multiple resistances among isolates of *S. maltophilia* associated with oilseed rape. However, as with most strains of clinical origin, all the isolates tested here remained susceptible to trimethoprim-sulfamethoxazole and minocycline.

Although other investigators have examined salad products for the prevalence of pathogenic bacteria (4), they did not attempt to isolate *S*. *maltophilia*. Another study may have underestimated the prevalence of the bacterium in these products since a selective medium was not used (5). We have shown in a clinical setting that the use of a medium selective for *S*. *maltophilia* improves the recovery of this bacterium (6). Furthermore, the optimal growth temperature of *S*. *maltophilia* is 30°C, and incubation at higher temperatures (7) may reduce the likelihood of recovering the bacterium from food products. We recommend that future studies of *S*. *maltophilia* in food products use a medium such as VIA and that cultures be incubated at 30°C.

Prepackaged, ready-to-eat salads, such as those examined in this study, are washed in chlorinated water before sale. This measure is clearly insufficient to remove *S*. *maltophilia* from these items, possibly because the bacterium may exist in biofilms in some of the components of these products. *S*. *maltophilia* is capable of forming biofilms on a number of materials (8). Alternatively, products may become contaminated from environmental sources in production plants after washing.

The importance of *S*. *maltophilia* in ready-to-eat salads, which are marketed in a manner that assumes the product does not need washing before consumption, is unknown; nevertheless the presence of the bacterium in these products serves to highlight recommendations that these items should be avoided by severely immunocompromised persons, especially those with neutropenia (9). Recently, Apisarnthanarak et al. (10), in a prospective study of hospitalized oncology patients, identified intestinal colonization with *S*. *maltophilia* in 4 (9.5%) of 41 patients, which emphasizes that foodstuffs may be a potential source of this bacterium for some patients. This is a preliminary study, however, and further studies are needed, in particular, molecular typing of food and human-associated isolates, to investigate the hypothesis that intestinal carriage of *S*. *maltophilia* may follow consumption of contaminated foodstuffs.

## References

[1] Denton M, Kerr KG. Microbiological and clinical aspects of infections associated with Stenotrophomonas maltophilia. Clin Microbiol Rev. 1998;11:57–80. 9457429 10.1128/cmr.11.1.57 PMC121376

[2] Berg G, Marten P, Ballin G. Stenotrophomonas maltophilia in the rhizosphere of oilseed rape—occurrence, characterization and interaction with phytopathogenic fungi. Microbiol Res. 1996;151:19–27. 10.1016/S0944-5013(96)80051-6

[3] Kerr KG, Denton M, Todd N, Corps CM, Kumari P, Hawkey PM. A new selective differential medium for isolation of Stenotrophomonas maltophilia. Eur J Clin Microbiol Infect Dis. 1996;15:607–10. 10.1007/BF01709373 8874082

[4] Lin C, Fernando SY, Wei C. Occurrence of Listeria monocytogenes, Salmonella spp., Escherichia coli and E. coli O157:H7 in vegetable salads. Food Contr. 1996;7:135–40. 10.1016/0956-7135(96)00019-9

[5] Denis C, Picoche B. Microbiologie des legumes frais predecoupes. Industrielle Alimentaire Agricoles. 1986;103:547–53.

[6] Denton M, Hall MJ, Todd NJ, Kerr KG, Littlewood JM. Improved isolation of Stenotrophomonas maltophilia from sputa of patients with cystic fibrosis using a selective medium. Clin Microbiol Infect. 2000;6:397–8. 10.1046/j.1469-0691.2000.00098.x 11168159

[7] Hagenmaier RD, Baker RA. A survey of the microbial population and ethanol content of bagged salad. J Food Prot. 1998;61:357–9. 9708311 10.4315/0362-028x-61.3.357

[8] Leriche V, Sibille P, Carpentier B. Use of an enzyme-linked lectinosorbent assay to monitor the shift in polysaccharide composition in bacterial biofilms. Appl Environ Microbiol. 2000;66:1851–6. 10.1128/AEM.66.5.1851-1856.2000 10788349 PMC101422

[9] Heard G. Microbial safety of ready-to-eat salads and minimally processed vegetables and fruits. Food Sci Technol Today. 2000;14:15–21.

[10] Apistharnthanarak A, Fraser VJ, Dunne WM, Little JR, Hoppe-Bauer J, Mayfield JL, Stenotrophomonas maltophilia intestinal colonization in hospitalized oncology patients with diarrhoea. Clin Infect Dis. 2003;37:1131–5. 10.1086/378297 14523780

